# Formal help-seeking among community-based Czech individuals with sexual interest in minors is associated with the perceived urgency of self-identified concerns

**DOI:** 10.3389/fpsyg.2025.1546102

**Published:** 2025-09-11

**Authors:** Lenka Martinec Nováková, Lucie Krejčová, Klára Bártová, Renáta Androvičová, Kateřina Klapilová

**Affiliations:** ^1^Department of Psychology and Life Sciences, Faculty of Humanities, Prague, Czechia; ^2^Center for Sexual Health and Interventions, National Institute of Mental Health, Klecany, Czechia

**Keywords:** barriers, children, mental health, paraphilia, prevention, therapy, pedophilia, sexual abuse

## Abstract

**Background:**

In the community, there are non-forensic individuals who experience sexual interest in minors and have specific mental health needs and concerns. If left unaddressed, these issues may contribute to problematic sexual behaviors. Although supportive treatment programs are emerging in Czechia, self-motivated help-seeking remains generally low in this at-risk population.

**Objective:**

To inform strategies for encouraging preventive service use, this study aimed to examine how past help-seeking experiences relate to factors that may influence the likelihood of seeking professional help, as identified in the literature.

**Methods:**

An online survey was conducted with a purposive sample of 97 community-based, predominantly male, non-forensic adults whose responses to hypothetical scenarios suggested a sexual interest in minors. The study investigated the relationship between self-reported past formal help-seeking behavior (any vs. none) and two key variables: perceived urgency of self-identified concerns and dismissive attitudes toward professional assistance. Analyses controlled for other forms of support sought.

**Results:**

Formal support sources were rarely approached. A significant association was found between low perceived urgency of self-identified concerns and the absence of formal help-seeking behavior (*β* = 0.33, *F*(1) = 6.34, *p* = 0.014).

**Conclusion:**

To promote professional service uptake among this difficult-to-reach population, it is essential to enhance mental health literacy across the broader community and to educate individuals about the role of psychological well-being in preventing problematic behaviors.

## Introduction

In recent years, researchers have become increasingly aware of the high prevalence of sexual interests in the general population that “go beyond the norm” and were often overlooked as supposedly “less common” (Santtila et al., 2015; Noorishad et al., 2019; Bártová et al., 2021; Savoie et al., 2021; Baier, 2024; Barker et al., 2025; Spada et al., 2025). According to the text revision of the fifth edition of the Diagnostic and Statistical Manual of Mental Disorders (DSM-V-TR) (APA, 2022), sexual interests may involve sexual fantasies, urges, or behaviors. If a person harbors “any intense and persistent sexual interest other than sexual interest in genital stimulation or preparatory fondling with phenotypically normal, physically mature, consenting human partners,” they are thought to exhibit paraphilia. In people who report not having any “intense and persistent” sexual interests, paraphilia would be defined as any sexual interest that is as strong as, or stronger than the above-mentioned normophilic interest. Moreover, some paraphilias are more accurately characterized by consistent patterns of sexual preference rather than by their intensity (APA, 2022, p. 780). Importantly, paraphilias are to be distinguished from paraphilic disorders. A paraphilic disorder refers to a paraphilia that either causes significant distress or functional impairment for the individual, or involves behaviors that have resulted in harm – or the potential for harm – to others. Thus, simply having a paraphilic interest does not automatically indicate a need for clinical attention or intervention (APA, 2022, p. 781).

Even though paraphilic interests as such may not necessarily be of concern in community-based non-forensic individuals (i.e., individuals who might or might not have offended and are not involved with the law enforcement and criminal justice system), they nevertheless tend to co-occur with specific psychological needs and problems. These include anxiety and depression, low self-esteem and self-acceptance, shame and stigma, suicidality, difficulties in romantic relationships, loneliness, maladaptive coping strategies, hypersexuality, diminished overall well-being, and a sense of hopelessness that discourages them from pursuing meaningful life goals (Cohen et al., 2020; Fox et al., 2022; Wibowo et al., 2022; Brown et al., 2023; Chronos et al., 2024; Lassche et al., 2024; Murphy, 2024; Barker et al., 2025; de Tribolet-Hardy et al., 2025; Konrad et al., 2025; Lievesley et al., 2025b; Yakeley et al., 2025; Zidenberg et al., 2025). Research shows that timely and sustained provision of adequate professional assistance improves clients’/patients’ psychosocial and psychosexual functioning (Barros et al., 2022; Clayton et al., 2022; Lätth et al., 2022; Lievesley and Harper, 2022; Piwowar et al., 2022; Beier et al., 2024; Goerlich, 2024; Hales et al., 2024; Heindl et al., 2024; de Tribolet-Hardy et al., 2025; Navrátil et al., 2025). On the other hand, unrecognized and unaddressed concerns may be associated with a risk of mental health deterioration, and the major psychological distress may in turn increase the likelihood of losing self-control and engaging in problematic sexual behaviors (Cohen et al., 2018; Mokros and Banse, 2019; Wild et al., 2020; Lampalzer et al., 2021; Chan, 2023; Chan and Myers, 2023; Swaby and Lievesley, 2023).

Although severe psychological, psychiatric, and neurological conditions may predict problematic sexual behaviors irrespective of paraphilia (Van der Molen et al., 2023; Babchishin et al., 2025; Warkentin et al., 2025), paraphilic interest can mediate the relationship between certain mental health issues and sexual offending (Engel et al., 2025). Also, paraphilic interests can independently serve as predictors of corresponding paraphilic behaviors (Seto et al., 2021; Joyal and Carpentier, 2022; de Roos et al., 2025; Lehmann et al., 2025; Reichert et al., 2025). Since some paraphilic interests would constitute criminal behavior if acted upon, they are commonly perceived as more “socially dangerous” and “high-risk” (Chatterjee, 2023; Požarskis and Požarska, 2023; Agapoff et al., 2024). One of them is pedophilia, [i.e., sexual interest in minors aged approximately 13 years or younger (APA, 2022, p. 794)]. “Pedophile” has been replaced by some academic researchers with the term “minor-attracted person” (MAP), which is intended to serve as a neutral and non-stigmatizing alternative (Farmer et al., 2024).

Nevertheless, the concordance between sexual interest and behaviors is rather weak for pedohebephilia [i.e., sexual interest in minors roughly under 15 years of age (Blanchard et al., 2009)]. For instance, in online samples, Seto et al. (2021) found a correlation of r = 0.275, and de Roos et al. (2025) in the full sample reported Kendall’s Tau-b correlations of 0.09 and 0.03 for pedophilia and hebephilia, respectively (0.23 and 0.17, respectively, in the subset of individuals who found the given paraphilic theme arousing). Although pedophilia-related sexual offenses tend to be conflated with hands-on (contact) acts of sexual abuse in lay people’s view (Glina et al., 2022), they far more often involve consumption of child sexual abuse material (CSAM) (Seto, 2019; Beier et al., 2024; Erkan et al., 2024). CSAM consumption is predicted by pedophilic interest (Lätth et al., 2025), and intensifies it even further (Paquette et al., 2022). Hence, there is a widespread public concern that it progresses to hands-on offending (Hunn et al., 2022; Langvik et al., 2024). According to Insoll et al. (2024), 42% of the participants admitted to seeking online contact with minors following their exposure to CSAM. Additionally, 58% expressed concern that their viewing of such material could escalate into real-world sexual encounters with either minors or adults. Still, the major concern in non-forensic individuals with pedophilic interest is the recidivism risk for viewing CSAM (von Franqué et al., 2023; Beier et al., 2024). The mental health issues (Kothari et al., 2021; Nurmi et al., 2024) and numerous repercussions associated with illegal expressions of this paraphilic interest (Jones et al., 2023; Kavanagh et al., 2023; Armitage et al., 2024; Kavanagh et al., 2024; Salter et al., 2024) are no less serious for CSAM-only offenders and their significant others. All of this suggests that early interventions, which could effectively promote mental well-being and encourage prevention and desistance in the non-forensic population, should be a public health priority (Price et al., 2024). The European Commission (2020) strategy for strengthening the response to sexual offending against minors recognizes a significant gap in secondary prevention initiatives across EU member states. It emphasizes the urgent need for tailored interventions targeting individuals at elevated risk (such as those with paraphilic disorders), alongside proactive strategies like digital outreach in collaboration with internet service providers.

In sum, there are many benefits to be gained from offering timely, targeted professional help to community-based individuals who may or may not consider themselves at risk of sexual offending and seek support in managing their sexual experiences and maintaining psychological well-being. These people frequently express a need for professional assistance yet encounter difficulties in accessing appropriate services through the “mainstream” healthcare and social work systems (Jimenez-Arista and Reid, 2023; Schaefer et al., 2023; Murphy, 2024; Lievesley et al., 2025b). In response to this recognized need for a public health approach (Cant et al., 2022), several European countries have implemented programs aimed at non-forensic individuals who are concerned about their sexuality (Beier et al., 2009; van Horn et al., 2015; Gibbels et al., 2019; Hallberg et al., 2019; Adebahr et al., 2021; Bellis et al., 2024). The Czech Republic has joined these efforts with the Parafilik (Paraphile) program relatively recently (Krejčová et al., 2021; Di Gioia and Beslay, 2023; Páv et al., 2024; Navrátil et al., 2025). The help-seeking environment in the Czech Republic represents a pioneering effort in establishing structured prevention strategies within the Central European region. Even though several similar initiatives already exist in the European Union offering help to non-forensic individuals with sexual interest in minors, for review see Di Gioia and Beslay (2023), their experience offers limited guidance when it comes to scaling or implementing similar measures at the national level. This is because essential aspects of service provision, such as the legal requirements for mandatory reporting, healthcare, social support, and crime prevention infrastructures, differ from country to country (Mathews, 2015; Klapilová et al., 2019).

Therefore, to ensure that the service offered reaches the target clientele and stays relevant to *their* treatment needs, which is essential for ensuring meaningful and effective therapeutic outcomes (Lievesley et al., 2023; Woodward et al., 2024; Lievesley et al., 2025a; Lievesley et al., 2025b), it is necessary to map the potential clients’ motivations, paths, and obstacles to formal help-seeking within the present national settings. To clarify, formal help-seeking is a problem-focused, planned behavior that involves interaction with a professional source that has a legitimate and specialized role in providing relevant support, counselling, and/or treatment (Cornally and McCarthy, 2011). It is frequently preceded by informal help-seeking (McCann and Lubman, 2018), which involves receiving support from social networks and community sources (e.g., significant others). Individuals from socially marginalized groups, such as those with stigmatized attractions, often find that it is safer to connect online with others who face similar challenges (Jones et al., 2021; Nielsen et al., 2022; Jimenez-Arista and Reid, 2023; Bekkers et al., 2024; Murphy, 2024).

In non-forensic contexts, help-seeking individuals are primarily guided by intrinsic motivations to address personal distress (Levenson and Grady, 2019b; Shields et al., 2020; Lievesley and Harper, 2022); hence, a major attitudinal barrier to formal help-seeking is a low perceived need for intervention. This is in agreement with treatment-seeking models such as the Behavioral Model of Health Services Use (Andersen, 1995; Andersen and Davidson, 2007) and Health Belief Model (Janz and Becker, 1984), which highlight the fact that people are generally unlikely to approach a health professional about a concern they themselves do not perceive as sufficiently pressing. This is particularly the case for people with pedophilic interest who often face a difficult dilemma: whether to reveal their concerns to trusted others and professionals in hopes of receiving support, while also confronting the real possibility of social rejection and exclusion (Fafejta, 2021; Lehmann et al., 2021; Combridge and Lastella, 2023; Chronos et al., 2024; Lawrence and Willis, 2024; Lehmann et al., 2024). There is a profound sense of stigma surrounding the public debate about pedophilia (Jara and Jeglic, 2021; Harper et al., 2022; McKillop and Price, 2023; Lehmann et al., 2024). Sexual interest in minors tends to be conflated with child sexual abuse in the public perception (Glina et al., 2022), media (Stelzmann et al., 2020, 2022; Ischebeck et al., 2024), and sometimes even in the literature (Zakaria, 2018; Kulik et al., 2021; Fernandez et al., 2023). The perceived “dangerousness” of people with pedophilia (Combridge and Lastella, 2023) makes the idea of them relieving their sexual desire (even in a noncriminal way) uncomfortable to many lay people (Lehmann et al., 2024) and professionals alike (Nematy et al., 2024). Although social stigma associated with pedophilic interests is relatively less frequent among professionals (Schmidt and Niehaus, 2022), some express discomfort or outright reluctance when it comes to treating even non-offending individuals (Levenson and Grady, 2019a; Bayram et al., 2021; Christophersen and Brotto, 2024). Hence, in non-mandated settings, concerns about professionals’ stigmatizing attitudes, misconceptions about pedophilia, and knowledge inaccuracies about mandatory reporting (Beggs Christofferson, 2019; Grady et al., 2019; Levenson and Grady, 2019a; Stephens et al., 2021; Walker et al., 2022) turn out to be major barriers to formal help-seeking (Grady et al., 2019; Jahnke et al., 2024). However, in countries where early intervention/primary prevention initiatives have been launched only recently, misgivings about service accessibility (which is an example of a structural barrier) may also be of relevance (Tenbergen et al., 2021; Jackson et al., 2022).

Self-referred help-seeking regarding paraphilia-related psychological needs in non-mandated settings is an under-researched topic in Central Europe. To understand specifically how Czech people with sexual interest in minors navigate the help-seeking context, we recruited a non-random, purposive sample of 97 community-based (predominantly male) non-forensic adults who reported high sexual arousal to hypothetical or imagined involvement with minors. In light of the existing evidence, we hypothesized that reports of past formal help-seeking would be positively predicted by disclosures to significant others and greater perceived urgency of self-identified concerns, and negatively by barriers reflecting dismissive attitudes towards professional assistance and perceptions of its poor accessibility.

## Materials and methods

### Sample and procedure

The target group of this study were male and female Czech-speaking community-based Czech citizens aged 18–80 years who expressed sexual interest in minors. They were recruited from two national pools of Czech respondents via the STEM/MARK sociodemographic agency^1^ in January—February 2020. This agency sources its respondents from the European national panel,^2^ which comprises 55,000 individuals, and the Dialog panel^3^ with 10,000 active members. Both the panels are run in compliance with the ethical codex of ICCP/ESOMAR.^4^ Only Czech nationals were targeted by the campaign. Deliberate quota sampling was employed, followed by purposive screening for selected paraphilic interests. The quotas involved region of residence, municipality size, gender, age, and education. Quotas were established on the basis of the most recent population census of the Czech Statistical Office at that time (CZSO, 2013). Responses were collected by means of a standardized online interview in the form of an online survey. This method was preferred due to the confidential nature of the survey, which helped preserve the respondents’ privacy. The current study was part of an umbrella project “Love and Intimacy in the Czech Population”; for other outputs, see, e.g., Marečková et al. (2022) and Zakreski et al. (2024). The present sample was nonetheless recruited with a different sampling procedure than the nationally representative sample, and the two samples did not overlap. We aimed to obtain about 100 complete responses for this particular study. To estimate the initial sample size, we utilized the findings of the study conducted by Bártová et al. (2021) on the prevalence of various paraphilic interests in the Czech Republic. The link to the survey was e-mailed to 5,422 members of the two survey panels. Out of all individuals contacted, 1,062 did not access the provided link. A total of 753 individuals clicked the link but did not complete the survey, 2,175 people did not meet the screening criteria related to paraphilic interests (see below), and 1,255 individuals were not included in the survey due to reaching the required sample size based on statistical power calculations and financial limitations. A total of 178 people met the screening criteria indicating interest in hypothetical sexual violence and/or pedophilic themes, of which 81 met the criteria for violent paraphilic interest but did not meet the criteria for pedohebephilic interest, and hence were not included in the present study. The final sample reported in the present paper comprised 97 respondents (78 male) aged 47.2 ± 14.5 (21–80) years. Participants who did not qualify based on the paraphilic interest criteria were filtered out and did not answer any additional questions.

**Figure 1:**
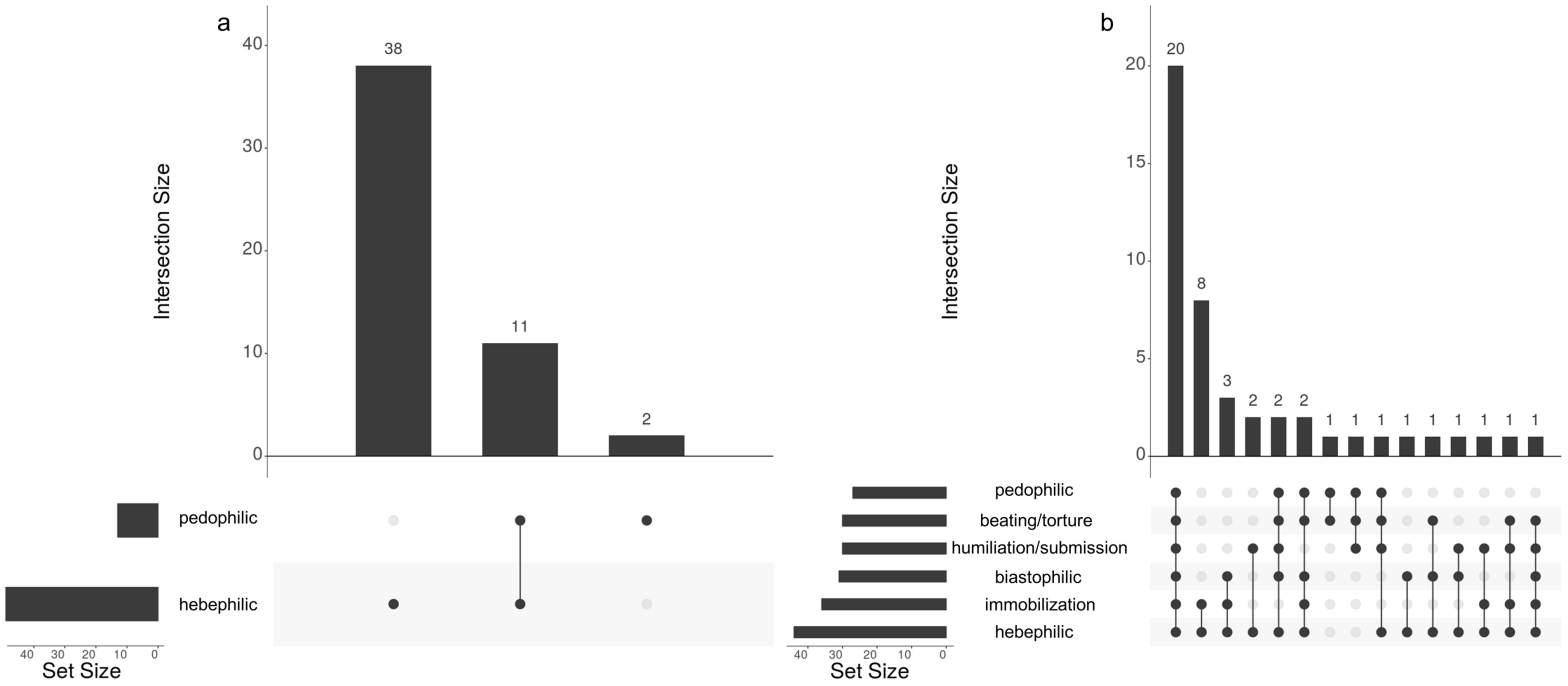
UpSet diagram of frequencies of screening paraphilic themes rated with a “4” or “5” and their combinations in the ME **(Panel a)** and MN **(Panel b)** group. Set size indicates how many times the given individual theme appeared across all responses within the group. Intersection size shows the absolute frequencies of a particular type of response featuring a single theme or their combinations. Empty intersections have been omitted from the plot.

Similar to several previous studies (Grundmann et al., 2016; Seto et al., 2021), sexual interest was assessed using ratings of sexual arousal in the hypothetical event of exposure to selected themes. It is important to emphasize that a person may experience strong sexual arousal in response to a particular theme without having any desire to act on it in real life. Pedophilic and hebephilic interest, respectively, were assessed with one item each, which were introduced as follows: *“In the next part of the survey you will be presented, besides common sexual activities and partners, with less usual sexual patterns that some people may find unpleasant. For others, however, they may represent a preference that they feel uncomfortable discussing. Please tell us how you feel about them. Provide candid answers using the following scale, where a rating of “1” stands for “definitely not” and a rating of “5” means “definitely.” The survey is anonymous, and the findings will only be used for scientific purposes. Does the idea of the following activities arouse you sexually?”* The respondents were then presented with two items representing the pedophilic (“Sexual contact with a minor under 12 years of age”) and hebephilic (“Sexual contact with a minor under 15, but over 12 years of age”) theme. These specific age categories were established to maintain consistency with the majority of prior studies, which employed similar groupings when examining patterns of sexual interest (e.g., Santtila et al., 2015; McPhail et al., 2019; Martijn et al., 2020). Additionally, to explore potential concomitant paraphilic interests, another four themes featured in the 10th revision of the *International Classification of Diseases* (World Health Organization, 2015) were presented, namely immobilization, biastophilia, humiliation/submission, and beating/torture; for definitions see Zakreski et al. (2024). A respondent was included in the final sample if they endorsed, i.e., rated with a “4” or “5”, the pedophilic and/or hebephilic theme. If these were the only themes endorsed, the participant was considered “minor-exclusive” (ME; *N* = 51, 52.6%). Those who endorsed any other paraphilic theme(s) in addition to the pedophilic/hebephilic one were labeled “minor non-exclusive” (MN; *N* = 46, 47.4%). The absolute frequencies of endorsements of the individual paraphilic patterns are shown in Figure 1.

Before proceeding to complete the survey, participants were asked to provide informed electronic consent, agreeing to participate in the survey. The survey took, on average, 46 min to complete (median time = 42 min). The online survey was available only in the Czech language and included items covering basic sociodemographic data, formal and informal help-seeking experiences, self-identified treatment targets and their perceived urgency, and attitudinal and structural barriers to help-seeking. All participants received the items in an identical sequence.

The study was approved by the Institutional Review Board of the National Institute of Mental Health, Approval No. 119/19. The process of data storage and anonymity assurance complied with the ethical codex of ICCP/ESOMAR (see text footnote 4). STEM/MARK awards participants with credits for completing surveys, which can later be redeemed for various rewards.

## Survey topics and measures

### Sociodemographic data

The respondents were asked to provide information on their age, gender, sexual orientation, educational background, size of place of residence, and relationship status. Sexual orientation was assessed on a 7-point Kinsey scale (ranging from 0 = “exclusively heterosexual” to 6 = “exclusively homosexual”) (Kinsey et al., 1948; Kinsey et al., 1953).

### Experiences with formal and informal help-seeking

**Figure 2:**
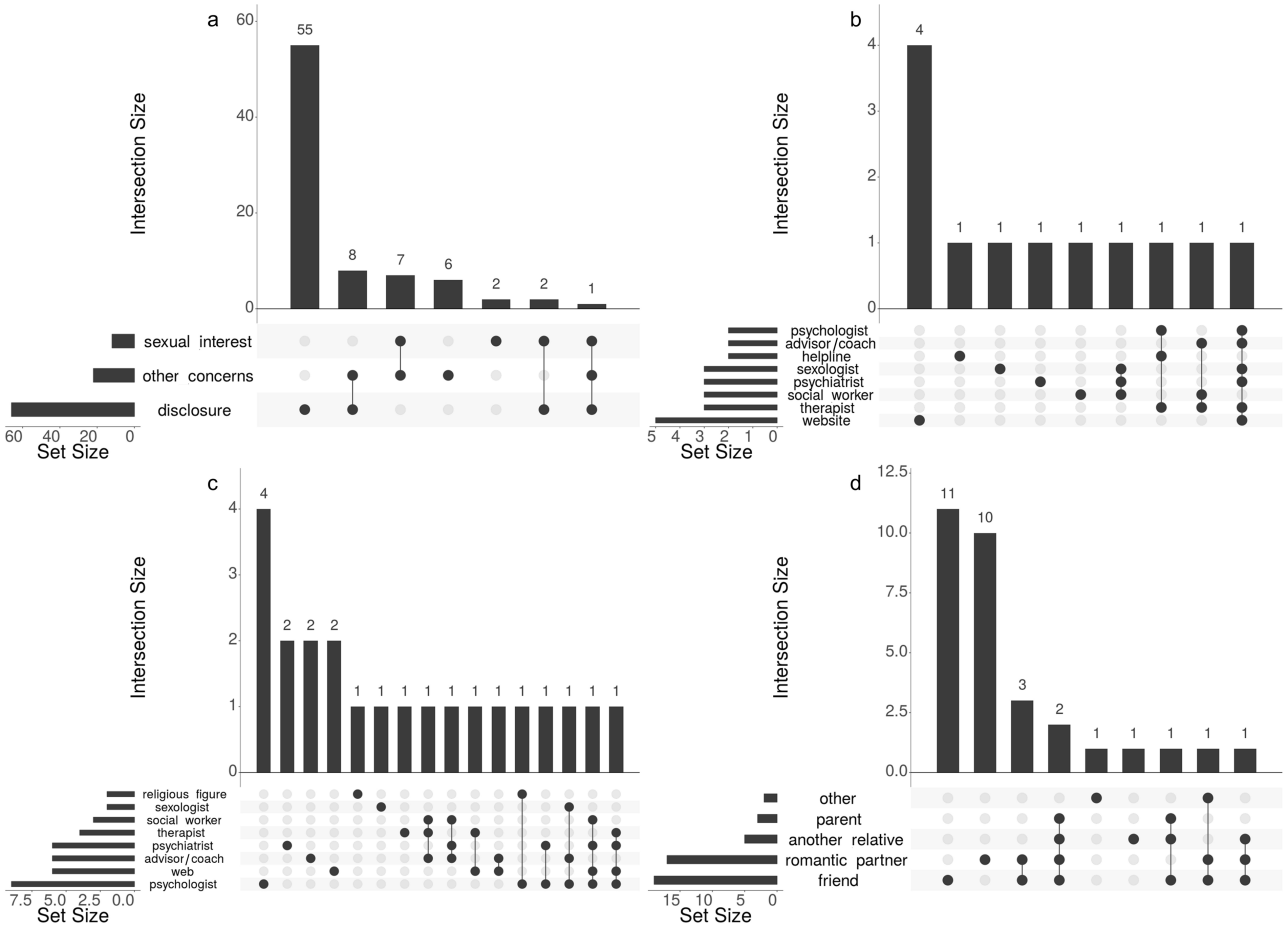
Use of sources of formal and informal support. **(Panel a)** Frequencies of formal help-seeking for sexual interests and other psychosocial concerns, and informal help-seeking (disclosures to significant others). **(Panel b)** Sources where formal help was sought for sexual interests. **(Panel c)** Sources where formal help was sought for other psychosocial concerns. **(Panel d)** Significant others that participants disclosed to about their sexual interest in minors. Not endorsed items are not displayed and empty intersections have been omitted from the plots.

The respondents were asked whether they had ever attempted to seek (1) formal help regarding their sexual interests (yes/no). Those who responded in the affirmative were consequently presented with a list of potential sources of formal help and support. The list was inspired by Levenson and Grady (2019b). The items that received non-zero endorsements are shown in Figure 2. Also, for each selected item, respondents were asked to indicate how helpful, in their view, the experience was (1 = “not at all helpful,” 2 = “somewhat helpful,” 3 = “very helpful”). Then they were instructed to indicate in the same manner whether they had ever sought (2) formal help about other psychosocial issues. To explore (3) disclosure and informal help-seeking with significant others, the respondents were asked whether their sexual interests were known to anyone (no one, romantic partner, parent, another relative, friend, other). All items were phrased in terms of “sexual interests” without any further specifications (such as “paraphilic” or “unusual”) to avoid use of language that might be perceived as presumptuous or judgmental.

### Self-identified concerns and their perceived urgency

Urgency of self-identified concerns was operationalized as a median rating of potential treatment targets, which were presented in a list that was inspired by Levenson and Grady (2019b) (see Table 1 for details). Participants were asked to rate each item in terms of perceived urgency on a five-point Likert-type scale anchored with “not a concern at all” (0) to “a very urgent concern” (4).

**Table 1:** Endorsements of items representing possible self-identified concerns and treatment targets in the minor-exclusive (ME) and non-exclusive (MN) groups.

	ME (*N* = 51)	MN (*N* = 46)	Total (*N* = 97)
Enhance quality of life	8	10	18
Anxiety and depression	8	9	17
Find a romantic/intimate partner	7	10	17
Get rid of unhealthy or unwanted ways of coping (e.g., watching porn)	5	8	13
Self-esteem	5	8	13
Concerns about future	6	5	11
Sexual frustration	4	7	11
Shame and stigma	3	6	9
Understand causes of sexual interests	4	5	9
Learn about one’s diagnosis	5	3	8
Identity issues	3	5	8
Adopt socially acceptable, desirable ways of coping	1	6	7
Deal with loss of sexual self	2	5	7
Reduce sexual attraction to minors	2	5	7
Manage sexual feelings	2	5	7
Disclosure and confidentiality issues	1	3	4
Enhance sexual attraction to adults	0	3	3

The frequencies represent the number of people in either group who rated the given item as being of urgent (“3”) or very urgent (“4”) concern.

### Attitudinal barriers to formal help-seeking

Attitudinal barriers to formal help-seeking were operationalized in two ways: (i) as a low degree of motivation to seek professional assistance, which was assessed with the *Therapy Motivation Scale* (TMS; Jahnke et al., 2015c), and (ii) a strong sense of social stigma towards people with sexual interest in minors, which was evaluated with the *Perceived Social Distance Scale* (PSDS) (Jahnke et al., 2015c).

#### Therapy motivation scale

The original purpose of the four-item TMS was to gauge the willingness of minor-attracted men to approach a professional about their sexual interest in minors. Agreement with statements is indicated using a seven-category response format (0 = “do not agree at all” to 6 = “completely agree”). An alteration that we made to the original measure was inclusion of an extra item assessing the respondents’ perceptions of professionals’ readiness to address their concerns. This was motivated by previous findings suggesting that minor-attracted people did not find the professionals’ input helpful and hence their experience with formal help-seeking was largely negative (Wagner et al., 2016; Levenson and Grady, 2019b). The extra item read as follows: “I believe that professionals are sufficiently trained to help me deal with my concerns.” The degree of agreement with the five statements was indicated by placing a mark along a seven-point Likert-type scale anchored with “completely disagree” (0) and “completely agree” (6). Reverse items were recoded so that higher scores represented greater willingness to seek formal help, and the individual scores were then added together to produce the total score, which could range between 0 and 30. Psychometric properties of TMS are not known in community-based people with sexual interest in minors. The scale was translated to Czech by Anna Pilátová and a back-translation was produced by LMN, as were the other measures, unless stated otherwise.

#### Perceived social distance scale

The six-item PSDS was originally designed to help assess the perceptions of minor-attracted persons of the societal stigma associated with sexual interest in minors. Participants are asked to indicate the degree of agreement with statements regarding the willingness of the general public in their country to be accepting towards non-offending minor-attracted persons in their workplace, neighborhood, circle of friends, or as random acquaintances, and the general public’s beliefs that such persons should be incarcerated or dead. The statements are introduced as follows: *“Please indicate how, in your belief, most people in* [the country of the participant’s residence] *would respond to these statements concerning people who are dominantly sexually interested in children but have never committed a crime. I believe that most people in* [the given country] *think that…”* A seven-category response format is used (0 = “do not agree at all” to 6 = “completely agree”). In the present study, we adapted the original measure to ask how, in the participant’s belief, most people in the Czech Republic would respond to statements concerning people whose sexual fantasies involve sexual violence against adults but who have never committed sexual offense. This was to gauge the respondents’ perceptions of the public sentiment regarding paraphilic fantasies without insinuating that the participant’s own sexual interests could be problematic, as might have been the case had we used the instructions in their original wording. Responses were given on a seven-point Likert-type scale anchored with “completely disagree” (0) and “completely agree” (6). Reverse items were recoded so that higher scores represented greater perceived stigma. The individual responses were added together to obtain a total score (theoretical range: 0–36), as per the original use of the measure (Jahnke et al., 2015b; Jahnke et al., 2015c). Retest reliability of the German version was found to be high [r = 0.89 with a test–retest interval of 1 week among 34 university students Jahnke and Hoyer (2017), cit. Sec. Jahnke (2018)]. It exhibited high internal consistency (*α* = 0.82) and convergent validity in a German general population online sample (Jahnke et al., 2015a).

### Attitudinal and structural barriers to help-seeking

Additionally, to further explore the formal non-help-seekers’ reasons for dismissing help, a list of attitudinal and structural barriers, inspired by Levenson and Grady (2019b), was presented to them. They were asked, “What are your reasons for not seeking help about your sexual interests?” and instructed to endorse all items that applied to them. The list is displayed in Table 2.

**Table 2:** Endorsements of attitudinal and structural obstacles and barriers to seeking help about sexual interest-related concerns in the minor-exclusive (ME) and non-exclusive (MN) formal non-help-seekers and in total.

	ME (*N* = 45)	MN (*N* = 40)	Total (*N* = 85)
Do not need any help with sexual interest-related issues (A)	31	32	63
Can control oneself and will not harm or offend (A)	10	5	15
Concerned about being reported to law enforcement authorities (A)	3	2	5
Worried that the professional would react negatively (A)	3	2	5
Concerned about unethical breaches of confidentiality (A)	1	2	3
Not sure how to find a competent practitioner (S)	1	1	2
Financial issues (S)	1	1	2
Commuting or time constraints (S)	0	0	0

A, attitudinal, S, structural barrier.

## Statistical analysis

IBM SPSS 24.0 software was used to run all statistical tests. Cohen’s d was computed with the ESCI software. The UpSet diagrams were produced with the UpSetR Shiny App^5^ developed by Lex et al. (2014). We checked the continuous variables for outliers, ascertained their normality, and mean-centered them. Associations between dichotomous variables were assessed with Chi square tests of independence and, to determine the effect size, the statistics were converted to Pearson’s product–moment correlation coefficient (r) after Rosenberg (2010). For related samples, these associations were computed with McNemar’s tests. 95% bias corrected and accelerated bootstrap confidence intervals (95% BCa CIs) are given in square brackets.

To investigate the factors contributing to formal help-seeking in the past, a categorical regression (CATREG) model was fitted. Past formal help-seeking for paraphilic interests (yes/no) was regressed on perceived urgency of self-identified concerns (that was operationalized as the median rating of potential treatment targets) and attitudinal barriers to formal help-seeking operationalized as the TMS and PSDS scores, respectively, controlling for disclosures to significant others (yes/no) and formal help-seeking for other psychosocial issues (yes/no). Scale variables were treated as numeric and were discretized by ranking, and nominal variables were treated as such. A random initial configuration was selected, as recommended when at least one variable is treated as nominal. PSDS and TMS scores were not significantly correlated in the practical or statistical sense (r = −0.01 [−0.24, 0.22], *p* = 0.912), meaning that multicollinearity was not an issue.

## Results

### Descriptive statistics

Table 3 shows the descriptive statistics in the ME and MN group. There were no significant differences between them in the sociodemographic variables. Furthermore, no within-group differences (gender or paraphilic pattern-related, i.e., hebephilic-only vs. pedohebephilic) or within-gender variation (i.e., ME vs. MN men and women) were found, either. The ME group nevertheless significantly differed from MN in that hebephilic-only endorsements prevailed, while the MN group was dominated by those who endorsed both the hebephilic and pedophilic patterns. ME respondents also found the two patterns significantly less sexually arousing.

**Table 3:** Descriptive statistics (mean ± SD [range], median or count) of age, education category, municipality population, relationship status and length, and sexual orientation as indicated on the Kinsey Scale in the minor-exclusive (ME) and non-exclusive (MN) group split up by gender.

	ME			MN		
	Male	Female	Total	Male	Female	Total
*N*	48	3	51	30	16	46
Age	46.0 ± 13.9 (22–80)	35.0 ± 9.5 (25–44)	45.3 ± 13.9 (22–80)	49.5 ± 15.0 (21–75)	48.8 ± 15.4 (25–72)	49.2 ± 15.0 (21–75)
Education						
Secondary without A level exam	11	2	13	9	8	17
Secondary with A level exam	24	0	24	13	5	18
Tertiary	13	1	14	8	3	11
Municipality population						
< 1,000	4	0	4	3	1	4
1,000–4,999	11	1	12	7	3	10
5,000–19,999	9	0	9	2	4	6
20,000–99,999	11	0	11	6	3	9
> 100,000	13	2	15	12	5	17
In a long-term relationship	30	2	32	21	10	31
Relationship length in years	11.7 ± 14.6 (0.2–51.8)	5.04 ± 1.24 (4.2–5.9)	11.3 ± 14.2 (0.2–51.8)	15.3 ± 14.0 (0.2–39.7)	10.7 ± 14.9 (0.2–39.3)	13.8 ± 14.2 (0.2–39.7)
Sexual orientation						
Exclusively heterosexual	38	1	39	22	14	36
Predominantly heterosexual, only incidentally homosexual	5	1	6	3	1	4
Predominantly heterosexual, but more than incidentally homosexual	0	1	1	2	0	2
Equally heterosexual and homosexual	1	0	1	3	1	4
Predominantly homosexual, but more than incidentally heterosexual	1	0	1	0	0	0
Predominantly homosexual, only incidentally heterosexual	1	0	1	0	0	0
Exclusively homosexual	2	0	2	0	0	0

Formal help-seeking for sexual interest-related concerns was only reported by 12 respondents (12.4%, 6 ME men, 3 MN men and women, respectively), as Figure 2, Panel a suggests. Almost half the help-seekers for sexual interest consulted some website(s) but approaching a professional was an exception rather than the rule (Panel b). There was almost a two-fold increase in the tendency to seek help for other reasons (*N* = 22) compared to help-seeking regarding sexual interest-related concerns (Panel c). As Panel d suggests, disclosures were mostly made to friends and romantic partners. Yet, a full 68% of respondents (*N* = 66) said their sexual interests were not known to anyone.

The list of suggested treatment targets (see Table 1) had limited relevance for over a half of the participants (57.7%, *N* = 56), who did not rate a single item from the list as urgent (“3”) or very urgent (“4”). Of the 41 (42.3%) respondents with any (very) urgent needs, 25 identified up to three (very) urgent treatment targets, and another 16 people had anywhere between four and eleven (very) urgent concerns. Between 52 and 79% of the respondents deemed any given treatment target completely irrelevant (i.e., rated it with a “0”). See the Supplementary material for more details.

As Table 2 reveals, the lack of perceived need for treatment was also reflected in the high endorsement frequency of the chief attitudinal barrier to help-seeking, i.e., belief that professional attention was not needed (*N* = 63).

Further descriptive statistics and explorations are given in the Supplementary material.

### Factors contributing to formal help-seeking

As Table 4 suggests, the only practically and statistically significant predictor was the median rating of potential treatment targets, with help-seekers rating the items as more urgent than non-help-seekers. With *β* < 0.5, the effect was small (Ferguson, 2009). The model was significant (*F*(5,91) = 8.27, *p* < 0.001) and explained nearly 1/3 of variability in formal help-seeking for sexual interests (R^2^ = 0.31, R^2^_adj_ = 0.275). Alternative models, in which perceived need for treatment was operationalized as the mean item rating, number of items rated as (very) urgent (i.e., rated with a “3” or “4”), or dichotomously as presence/absence of any (very) urgent needs, yielded similar results.

**Table 4:** Mean ± SD for the continuous and absolute (relative) frequencies for categorical predictors; and βs, F-statistics and *p*-values for the categorical regression (CATREG) of past help-seeking for sexual interest-related concerns on perceived urgency of self-identified concerns (operationalized as a median rating of treatment targets), Therapy Motivation Scale (TMS) and Perceived Social Distance Scale (PSDS) scores, and the binary variables of informal disclosures and formal help-seeking for other psychosocial issues.

	Mean ± SD	*N* (%)	β	F	*p*
Median treatment target rating	0.5 ± 1.0		0.33	6.34	0.014
TMS	13.1 ± 5.7		0.16	3.68	0.058
PSDS	18.7 ± 8.9		0.03	0.08	0.772
Disclosure		31 (32%)	0.16	3.22	0.076
Help-seeking for other mental health issues		22 (22.7%)	0.20	3.37	0.070

## Discussion

The aim of the present study was to examine to what extent perceived urgency of self-identified concerns, informal disclosures, and attitudinal barriers relate to reports of past formal help-seeking behavior for sexual interest-related concerns, controlling for help-seeking for other psychosocial issues. We hypothesized that past formal help-seeking for sexual interests would be linked to greater perceived urgency of self-identified concerns, operationalized as higher rated urgency of a set of treatment targets, occurrence of informal disclosures, and a lower degree of attitudinal barriers.

We found that formal help-seeking in the past was significantly predicted by a greater degree of pressing self-identified psychosocial needs, as reported in the present. Conversely, among non-help-seekers, the most prominent reason formal help was not sought was the perception that professional assistance was not urgently needed. Our results underscore the significance of the “need factors” – particularly the subjectively perceived need—as outlined in the influential Behavioral Model of Health Services Use (Andersen, 1995; Andersen and Davidson, 2007). The model identifies three primary categories of influencing factors, namely predisposing, enabling, and need factors, and it is the interplay among these three that ultimately determines whether an individual seeks professional help or not. Predisposing factors encompass demographic characteristics, social determinants including education, occupation, and ethnicity, and psychological dimensions, particularly health-related beliefs and literacy. Enabling factors refer mainly to financial (e.g., available income, out-of-pocket expenses) and organizational aspects (e.g., having a regular healthcare provider, service quality, waiting times, and logistical considerations). Finally, the model states that healthcare utilization depends on an individual’s personal recognition of the need for care and their own interpretations of their health status (i.e., perceived need), which may stand in contrast to evaluated need, which is based on clinical judgments and objective assessments made by healthcare professionals regarding the necessity for medical intervention. Research involving community-based, non-forensic populations has consistently shown that the motivation to seek help often stems from unmet perceived needs that significantly impact the individual’s overall well-being, such as anxiety and depression, low self-esteem, suicidal ideation, and loneliness (Levenson and Grady, 2019b; Stevens and Wood, 2019; Shields et al., 2020; Lievesley et al., 2023; Chronos et al., 2024; Insoll et al., 2024; Lievesley et al., 2025b). Other models employed to explain healthcare utilization, such as the Health Belief Model (Janz and Becker, 1984), also acknowledge the critical role of the individual’s subjective assessment of their situation. According to the Health Belief Model, a person’s decision to seek care is influenced by how vulnerable they believe they are to potential health complications if the condition is left untreated, as well as by their perception of how much the illness disrupts their daily life. Critically, an individual’s self-assessment of their condition may disregard factors that researchers and clinicians consider significant—such as sexual interests – unless those factors have a noticeable impact on their daily functioning (e.g., Lievesley et al., 2025b).

Consequently, there may be a considerable disconnect between the treatment goals prioritized by professionals – often centered on managing heightened risk of offending – and the objectives held by the client (Houtepen et al., 2016; Levenson et al., 2017). In non-mandated help-seeking settings, individuals who approach professionals for help are typically driven by internal motivations to alleviate their own psychological distress (Levenson and Grady, 2019b; Shields et al., 2020; Lievesley and Harper, 2022), not by the presence of “uncommon” sexual interests. Clients, both self-referred and court-ordered, may not readily accept or internalize risk reduction as a key objective of the treatment (Levenson, 2011; Mann et al., 2013; Barroso et al., 2019; Carrola, 2023). The lower levels of internalization may be reflected in the presence of stronger offense-supportive cognitions (Zakreski et al., 2024), with individuals less motivated to seek therapy tending to display higher levels of offense-supportive cognitions (Jahnke et al., 2015c). In fact, some of our respondents may not even have interpreted their endorsement of a hypothetical scenario involving minors as potentially indicative of a problematic sexual interest. Throughout the survey, participants were invited to share their views on various aspects of help-seeking, and six of them chose to elaborate on their reasons for not seeking formal help. One person misinterpreted the opportunity as an incentive to comment on their sexual orientation, saying, *“There is no need to seek help for bisexuality.”* Another participant stated that they did not perceive any issues related to their sexuality that would warrant seeking formal help: “*I do not have any problems sexually, maybe I’m a bit shy, but I do not need professional help for that.”* Yet another respondent’s reply suggested that they viewed their sexual interests as either insignificant or unlikely to affect their behavior in a concerning way: *“I do not believe any of my preferences would compel me to engage in illegal behavior.”* Nonetheless, two other respondents appeared to recognize that their endorsement of a theme involving minors might indicate a sexual interest that could be viewed as cause for concern. The defensive tone of their responses suggested a sense of discomfort or mistrust regarding what they anticipated from engaging with professionals. One of the respondents raised the subject of the immutability of pedophilic/hebephilic preferences and indirectly voiced concern that their own, if disclosed, might be targeted by conversion therapy: *“I do not think an expert would be able to figure it out. Sexual preference is something that is encoded in our brains. It is like someone liking red and being forced to like blue.”* The other participant’s response suggested that they perceived intervention as a means of enforcing societal norms or exerting social control: *“I reckon it’s only a matter of time before such people are compulsorily ‘supervised’.”* Finally, one respondent simply mentioned that they were shy (presumably to seek help about sexual issues).

It is possible that the current findings are subject to bias, as some individuals in the original sample [i.e., before filtering out those with low sexual arousal ratings to pedo(hebe)philic theme(s)] may have felt that endorsing themes involving minors could carry some negative implications and therefore chose not to respond candidly. If that were the case, the proportion of *non*-help-seekers for sexual interests in the current sample might be an underestimation, as those individuals would also likely either deny having any needs related to their sexual preferences or downplay their significance. Even though the survey items were deliberately worded to avoid language that could be interpreted as presumptive or judgmental, the stigma of pedophilia is so deeply ingrained in the public consciousness that it often hinders open and honest dialogue (Lawrence and Willis, 2021; Lehmann et al., 2021; Glina et al., 2022). Moreover, as illustrated by the participant quotes above, there seems to be a degree of mistrust toward therapeutic goals as perceived to be defined by the therapists. Specifically, conversion therapy has gained considerable negative attention in the public eye over the decades (Davison and Walden, 2024), and even though most practitioners now denounce such methods as unethical, ineffective, and harmful (Cantor, 2018; Alempijevic et al., 2020; Andrade and Campo Redondo, 2022; Nematy et al., 2024), among non-experts, they still seem to linger in the spotlight. In consequence, people with paraphilic interests may be apprehensive about how a therapist might respond to their disclosure and so they choose to avoid the situation altogether (Levenson and Grady, 2019b). Conversely, positive attitudes towards formal help-seeking are linked to greater familiarity with intervention and reduced expectation of a negative response from the therapist (Jahnke et al., 2024). Therefore, intervention ought to be presented to clients as a supportive and empowering process, not simply a means of ‘prevention’. It is important for professionals as well as potential clients to embrace the idea that interventions should not seek to alter sexual interests but, rather, provide people with strategies to navigate their experiences in ways that align with their personal values and promote psychological well-being (Lievesley et al., 2023; Lievesley et al., 2025a; Lievesley et al., 2025b).

Despite dismissing formal help as unnecessary, a significant number of participants in our sample demonstrated a clear need for psychological support, as indicated by their endorsements of pressing mental health issues. Importantly, a full third of those who felt they did not need any help (*N* = 21) indicated having at least one urgent mental health-related concern. Even though it might appear that people with mental health challenges are managing without professional help, we must not be lulled into accepting this as an adequate or sustainable solution. Addressing the specific needs of these individuals aligns with the fundamental aim of health and social care interventions—to offer respectful and compassionate support to all who seek it (Levenson et al., 2020). Moreover, it contributes to fostering protective factors that encourage healthy sexual behaviors that do not put oneself or others at risk (de Vries Robbé et al., 2015; Cohen et al., 2018; Lievesley and Harper, 2022). Emerging evidence suggests that supportive treatment programs tailored for non-forensic community-based individuals with paraphilic interests and psychological needs may serve as an effective strategy for preventing problematic sexual behaviors (Gibbels et al., 2019; Parks et al., 2020; Adebahr et al., 2021; Tenbergen et al., 2021; Beier et al., 2024; Heindl et al., 2024). However, as noted above, professional training initiatives aimed at supporting help-seeking individuals with paraphilic interests should emphasize a client-centered approach to risk assessment and treatment (Levenson et al., 2020; Lievesley et al., 2025a). Also, public discussions should shift toward highlighting therapeutic strategies that foster dignity, empowerment, and well-being, rather than concentrating predominantly on managing risks (Chronos et al., 2024). Client-centered interactions enhance service satisfaction, foster a strong therapeutic alliance, promote adherence to treatment, and ultimately contribute to better health outcomes (Robinson et al., 2008). Improving the quality of interventions for individuals with paraphilic interests can also help increase engagement with available services (Lasher and Stinson, 2017; Levenson et al., 2020; Lievesley et al., 2025a). This approach also aligns with the current EU strategy for a more effective fight against child sexual abuse proposed by the European Commission (2020).

In sum, the key to effective service provision to people with paraphilic interests in non-mandated settings is acknowledging that their motivations are as varied and personal as those of any other client seeking mental health support (Chronos et al., 2024). In terms of help-seeking for mental health issues, key obstacles include a low personal recognition of the need for support, unfavorable views toward treatment (Andrade et al., 2014), and limited mental health literacy, which hinders both the identification of problems and the acknowledgment of the need for care (Mojtabai et al., 2011; Jorm, 2012). Furthermore, stigma surrounding mental health concerns acts as a major obstacle to help-seeking, because a sense of shame and fear of being labeled discourage people from pursuing mental health support (Clement et al., 2015; Schnyder et al., 2017). Thus, successful approaches to increase formal help-seeking in people with self-driven incentives include population-wide mental health literacy campaigns and destigmatization (Xu et al., 2018). There is solid evidence that higher levels of mental health literacy are associated with more favorable attitudes toward seeking help and a greater willingness to pursue mental health support (Waldmann et al., 2020; Özparlak et al., 2023; Gündoğdu et al., 2024; Lien et al., 2024). Increasing perceived need for treatment through mental health literacy campaigns may thus encourage people who experience mental health issues to approach professionals more readily (e.g., Drane et al., 2023), which is how sexual interests may come to be co-addressed, although they may not be the original or primary reason for seeking formal help. Furthermore, destigmatization of mental health concerns is also needed, as even short-term interventions aimed at reducing stigma lead to more positive attitudes toward seeking formal help (Howard et al., 2018). Nevertheless, the positive effects tend to diminish over time, suggesting that follow-up sessions or continued engagement in mental health-related activities may be necessary to sustain improvements in help-seeking behavior (Shahwan et al., 2020). Community-wide destigmatization of sexual interest in minors, however, remains a highly contentious topic. On the one hand, the tendency to equate sexual preference for minors with actual acts of abuse intensifies the stigma directed at individuals who experience such attractions but do not offend. This may negatively affect their mental health, discourage them from seeking professional support, and, eventually, may inadvertently heighten the risk of harmful behavior by isolating them from preventive resources (Lawrence and Willis, 2021; Jahnke et al., 2024; McPhail, 2024). Advocates of public anti-stigma interventions propose that transitioning from a societal mindset rooted in fear and suspicion to one grounded in empathy and informed understanding could meaningfully enhance the quality of life and psychological well-being of individuals who experience these attractions and eventually promote offense-free lifestyles (Lawrence and Willis, 2021; Harper et al., 2022; Lawrence and Willis, 2022, 2023; McKillop and Price, 2023). Yet, on the other, some researchers (e.g., Farmer et al., 2024) argue that it is feasible to offer compassionate and effective therapeutic support to individuals with pedophilic tendencies without aiming for a broader societal acceptance or “normalization” of pedophilia. While further research is necessary to fully resolve this debate, current findings indicate that fostering self-acceptance and reducing internalized stigma are critical components of effective therapy for non-offending individuals with sexual attractions to minors. Developing a sense of self-acceptance has been recognized as a key therapeutic objective in addressing internalized stigma and shame (Levenson et al., 2020). Moreover, many of these individuals view coming to terms with their sexual preferences as essential not only for maintaining their mental health but also for strengthening their commitment to self-regulation – often taking pride in their ability to manage their interests responsibly (Levenson and Grady, 2019b; Stevens and Wood, 2019; Jones et al., 2021).

The broader implication of our study is that improving mental health literacy across the broader community is essential to reaching this population for intervention. By raising awareness about the connection between psychological well-being and ongoing commitment to remaining offense-free, we can help create conditions that encourage these people to seek professional support. However, our findings should be interpreted with caution, as they are based on responses from an online sample and may not be fully generalizable to the broader population of community-dwelling individuals with sexual interests in minors. Yet it is equally important to highlight the distinctiveness of our study in successfully engaging a population that has been largely overlooked in prior research. Specifically, our current understanding of formal help-seeking for sexual interest-related concerns is predominantly informed by research with people involved with the criminal justice system, persons already receiving care within the established healthcare framework, and self-identified pedophiles, which may not accurately reflect the experiences or needs of people with paraphilic interests in the broader community. While the online mode of contact likely leads to the exclusion of individuals without easy internet access, it can, on the other hand, increase the feeling of anonymity and encourage individuals to reveal their preferences more freely.

Further, the study relied exclusively on self-report measures, which are inherently susceptible to social desirability bias, particularly in research involving stigmatized topics such as sexual interest in minors. While anonymity was preserved, we cannot rule out the possibility that participants underreported or misrepresented their experiences. Additionally, some of the instruments used (e.g., PSDS and TMS) have not been validated for use with non-forensic individuals who do not self-identify as pedophilic. This raises questions about the construct validity of these measures in our sample and may partly explain the lack of significant associations between the attitudinal barriers and help-seeking behavior for sexual interests. The retrospective nature of the data further limits our ability to draw firm conclusions, as participants’ motivations and perceptions may have changed over time.

## Conclusion

The present study explored help-seeking behaviors and treatment needs among individuals with sexual interest in minors, assessed by means of subjective arousal ratings to hypothetical scenarios. Despite the presence of mental health concerns, formal help-seeking for sexual interest-related issues was rare. Most participants had never disclosed their interests, and when they did, it was typically to close personal contacts rather than professionals. This reluctance was mirrored in the high prevalence of attitudinal barriers, particularly the belief that professional help was unnecessary. The perceived urgency of treatment needs emerged as the only significant predictor of formal help-seeking, though the effect size was modest. Notably, over half of the sample found the proposed treatment targets irrelevant. These findings underscore the role of self-assessed urgency in motivating help-seeking. Enhancing mental health literacy and reducing internalized stigma may be key to increasing engagement with professional support in community-based non-forensic individuals with paraphilic interests. Future research should explore how to better align therapeutic offerings with the lived experiences of this population and investigate strategies to foster earlier, voluntary engagement with preventive services.

## Data Availability

The raw data supporting the conclusions of this article will be made available by the authors, without undue reservation.

## Footnotes

^1^ www.stemmark.cz

^2^ https://www.nationalpanel.eu/

^3^ https://dialog.stemmark.cz/

^4^ https://www.esomar.org/

^5^ https://gehlenborglab.shinyapps.io/
