# Supplementary material for: Formal help-seeking among community-based Czech individuals with sexual interest in minors is associated with the perceived urgency of self-identified concerns
Source: Front Psychol. 2025 Sep 11;16:1546102. doi: 10.3389/fpsyg.2025.1546102 (PMC12460231; doi:10.3389/fpsyg.2025.1546102)
Supplement: Supplementary file 1 [file Presentation_1.pdf]

Supplementary Material for:

Formal help-seeking among community-based Czech individuals with sexual interest in minors is associated with the perceived urgency of self-identified concerns

Lenka Martinec Nováková[1,2,*], Lucie Krejčová[2,1], Klára Bártová[2,1], Renáta Androvičová[2,1], and Kateřina Klapilová[2,1]

[1] *Department of Psychology and Life Sciences, Faculty of Humanities, Pátkova 2137/5, 182 00 Praha 8 – Libeň, Czechia*

[2] *Center for Sexual Health and Interventions, National Institute of Mental Health (NIMH-CZ), Topolová 748, 250 67 Klecany, Czechia*

[*] Corresponding author, email: lenka.novakova@nudz.cz, martinel@vscht.cz

Additional Explorations

Participants

The ME group composition was dominated by respondents who endorsed (i.e., rated with a "4" or "5") the hebephilic pattern only (N = 38, 75%, 37 males). In contrast, the majority of the MN participants endorsed all six screening paraphilic patterns (N = 20, 43%). Thus, the ME and MN groups differed in the proportion of participants who endorsed solely the hebephilic pattern vs. the hebephilic AND pedophilic patterns, $\chi^2(1) = 11.54$, $p < 0.001$. Also, the ME and MN groups differed in their median ratings of paraphilic patterns. Compared to the ME participants, the MN respondents found both the pedophilic ($\chi^2(4) = 17.92$, $p < 0.001$; median four vs. two) and hebephilic ($\chi^2(3) = 9.05$, $p < 0.05$; median five vs. four) patterns significantly more sexually arousing. There were no statistically significant differences in the rating of any other paraphilic theme.

Experiences with Formal and Informal Help-Seeking

Formal help was seldom sought regarding sexual interests. A total of 12 respondents (12.4%, 6 ME men, 3 MN men and women, respectively) indicated they had tried to seek formal help in the past about concerns related to their sexual interests. The low counts of help-seekers prevented exploration of between- and within-group differences with statistical tests. Still, visual inspection of absolute frequencies suggested that in the ME group, all but one help-seeker endorsed the hebephilic pattern only. On the other hand, in the MN group, all help-seekers endorsed both the hebephilic AND pedophilic patterns. The pattern of sources from which help was sought regarding sexual interest-related concerns and other mental health issues was rather varied. Almost half the help-seekers for sexual interest consulted some website(s). Direct contact with professionals was rare. When each respondent's median satisfaction with all of

their past help-seeking encounters was computed, it transpired that seven of them found the experience “somewhat helpful”, three felt that their help-seeking efforts turned out to be fruitless (median rating = 1), and two found the experience “very helpful” (median rating = 2.5 to 3). Eight respondents who had attempted to seek help for their sexual interest-related concerns had also done so for other issues. It was found that there was almost a two-fold increase in the tendency to seek help for other reasons (N = 22) compared to help-seeking regarding sexual interest-related concerns. Help-seeking for other issues was reported by nine (all male) respondents in the ME group and 13 (nine male) in the MN group.

Disclosures to significant others were significantly linked to formal help-seeking for sexual interest-related issues ($\chi^2(1) = 9.52$, $p = 0.002$, Cramer’s V = 0.347) as well as for other mental health concerns ($\chi^2(1) = 8.09$, $p = 0.004$, Cramer’s V = 0.315). Specifically, nine out of the 12 help-seekers (75%) for sexual interest-related issues said they had a close person they could talk to about their sexual interests. The proportion of disclosures among help-seekers for other concerns was similar (13 out of 22, 59.1%). Disclosures were mostly made to friends and romantic partners. Yet, a full 68% of respondents (N = 66) said their sexual interests were not known to anyone.

Perceived Urgency of Self-Identified Concerns

To assess each respondent’s perceived need for treatment, we produced a median from the 17 individual ratings, with higher scores suggesting a greater sense that professional assistance was needed. The median rating was significantly associated with formal help-seeking for sexual interests and other mental health concerns ($\rho = 0.35$, $p < 0.001$ and $\rho = 0.26$, $p = 0.012$, respectively) as well as informal disclosures ($\rho = 0.25$, $p = 0.016$), but not with PSDS or TMS scores ($|\rho| < 0.1$, $p = 0.500$).

Over half of the participants (57.7%, N = 56) did not rate a single item from the list with a “3” or “4”, meaning that they did not view any given treatment target as urgent/very urgent. Twenty-five respondents identified up to three urgent treatment targets, and 16 people had anywhere between four and eleven pressing concerns. From the viewpoint of the individual items as units of analysis, this is also reflected in the fact that each item’s median rating across the respondent sample was zero. Specifically, between 52% and 79% of the respondents deemed any given treatment target completely irrelevant (i.e., rated it with a “0”).

In the respondents who did report any urgent needs (42.3%, N = 41), the pattern of endorsed treatment targets was highly diverse and idiosyncratic. However, the most pressing concerns were quality of life, anxiety and depression, and forging a romantic companionship (endorsed by 18, 17, and 17 respondents, respectively). The number of self-identified concerns was linked in a practically and statistically significant way to past help-seeking concerning sexual interest-related issues ($\rho = 0.24$ [0.01, 0.45], $p = 0.020$) and other concerns ($\rho = 0.22$ [-0.02, 0.44], $p = 0.031$). However, it was not associated with disclosures ($\rho = 0.11$ [-0.11, 0.31], $p = 0.293$), PSDS ($\rho = 0.12$ [-0.08, 0.32], $p = 0.256$), or TMS scores ($\rho < 0.01$ [-0.19, 0.20], $p > 0.900$).

Attitudinal and Structural Barriers to Formal Help-Seeking

Respondents who indicated that they had not attempted to seek formal help regarding their sexual interests (N = 85) were asked why not. The two most frequently endorsed items reflected the attitudinal barriers, i.e., belief that professional attention was not needed (N = 63, of which 31 were in the exclusive group) and the belief that they could control their behavior and would not harm anyone or offend sexually (N = 15, 10 in the exclusive group). Endorsements of items representing structural barriers (e.g., poor accessibility of counselling

and/or treatment services) were rare. Respondents who believed that they did not need any professional help did *not* exhibit lower median ratings of potential treatment targets, lower TMS scores, or higher PSDS scores. They nevertheless reported fewer urgent treatment targets (i.e., those rated with a “3” or “4”; $\rho = -0.40$ [-0.59, -0.19], $p < 0.001$). In those who believed that professional assistance was unnecessary (N = 63), there were nine help-seekers for other mental health concerns (14.3%). In contrast, among the participants who did not endorse this attitudinal barrier (N = 22), there were five (22.7%). The ratios of informal disclosures were nevertheless similar (16/63 vs. 6/22, i.e., 25.4% vs. 27.3%).

Therapy Motivation Scale

Help-seekers in general exhibited a lower degree of attitudinal barriers in terms of treatment willingness, i.e., they were more motivated than non-help-seekers to approach a professional, as indicated by their higher TMS scores. This was true for help-seeking regarding sexual interest-related concerns ($t(95) = -2.23$, $p = 0.028$, mean difference = -3.84 [-7.26, -0.42], $d = 0.73$, 71.5% overlap), other concerns ($t(95) = -2.35$, $p = 0.021$, mean difference = -3.17 [-5.85, -0.49], $d = 0.61$, 76% overlap), as well as disclosures to significant others ($t(95) = -2.36$, $p = 0.020$, mean difference = -2.86 [-5.27, -0.46], $d = 0.51$, 79.9% overlap). In terms of seeking help for sexual interest-related issues, the participants’ motivation to do so was linked to their appraisal of the median helpfulness of past formal help-seeking experiences in a practically (but not statistically) significant way ($r = 0.33$ [-0.32, 0.82], $p = 0.296$, N = 12). There were no significant between-group or gender differences in the TMS scores. However, within the non-exclusive group, with a median TMS score of 15, pedohebephilic individuals (N = 25) were more motivated than the hebephilic-only respondents (median = 10, N = 19) to approach a professional ($t(42) = -2.89$, $p = 0.006$, mean difference = -4.64 [-7.89, -1.40], $d = 0.86$, 66.7%).

Similar computations in the exclusive group were not possible due to the low N of pedohebephilic respondents.

Perceived Social Distance Scale

Respondents who had disclosed to a significant other about their sexual interest had higher PSDS scores than those who had not ($t(95) = -2.73$, $p = 0.008$, mean difference = -5.10 [-8.81, -1.39], $d = 0.61$, 76% overlap). Still, formal help-seekers for sexual interests or other mental health issues did not differ on this measure. PSDS scores were significantly negatively related to median satisfaction with overall help-seeking experiences in a practical but not statistical way ($r = -0.53$ [-0.91, 0.12], $p = 0.075$, $N = 12$). There were no differences between the exclusive and non-exclusive group, men and women, or between pedohebephilic and hebephilic-only respondents within the non-exclusive group. PSDS scores did not correlate with TMS scores practically or statistically significantly.
